# Fast Nonnegative Matrix Factorization Algorithms Using Projected Gradient Approaches for Large-Scale Problems

**DOI:** 10.1155/2008/939567

**Published:** 2008-07-06

**Authors:** Rafal Zdunek, Andrzej Cichocki

**Affiliations:** ^1^Instiute of Telecommunications, Teleinformatics and Acoustics, Wroclaw University of Technology, Wybrzeze Wyspianskiego 27, 50-370 Wroclaw, Poland; ^2^Laboratory for Advanced Brain Signal Processing, Brain Science Institute RIKEN, Wako-shi, Saitama 351-0198, Japan; ^3^Institute of Theory of Electrical Engineering, Measurement and Information Systems, Faculty of Electrical Engineering, Warsaw University of Technology, 00-661 Warsaw, Poland; ^4^Systems Research Institute, Polish Academy of Science (PAN), 01-447 Warsaw, Poland

## Abstract

Recently, a considerable growth of interest in projected gradient (PG) methods has been observed due to their high efficiency in solving large-scale convex minimization problems subject to linear constraints. Since the minimization problems underlying nonnegative matrix factorization (NMF) of large matrices well matches this class of minimization problems, we investigate and test some recent PG methods in the context of their applicability to NMF. In particular, the paper focuses on the following modified methods: projected Landweber, Barzilai-Borwein gradient projection, projected sequential subspace optimization (PSESOP), interior-point Newton (IPN), and sequential coordinate-wise. The proposed and implemented NMF PG algorithms are compared with respect to their performance in terms of signal-to-interference ratio (SIR) and elapsed time, using a simple benchmark of mixed partially dependent nonnegative signals.

## 1. Introduction and Problem Statement

Nonnegative matrix factorization (NMF) finds such nonnegative factors (matrices) **A** = [*a*_*ij*_] ∈ ℝ^*I*×*J*^ and **X** = [*x*_*jt*_] ∈ ℝ^*J*×*T*^ with a *a*_*ij*_ ≥ 0, *x*_*jt*_ ≥ 0 that **Y** ≅ **AX**, given the observation matrix **Y** = [*y*_*it*_] ∈ ℝ^*I*×*T*^, the lower-rank *J*, and possibly other statistical information on the observed data or the factors to be estimated.

This method has found a variety of real-world applications in the areas such as blind separation of images and nonnegative signals [1–6], spectra recovering [7–10], pattern recognition and feature extraction [11–16], dimensionality reduction, segmentation and clustering [17–32], language modeling, text mining [25, 33], music transcription [34], and neurobiology (gene separation) [35, 36].

Depending on an application, the estimated factors may have different interpretation. For example, Lee and Seung [11] introduced NMF as a method for decomposing an image (face) into parts-based representations (parts reminiscent of features such as lips, eyes, nose, etc.). In blind source separation (BSS) [1, 37, 38], the matrix **Y** represents the observed mixed (superposed) signals or images, **A** is a mixing operator, and **X** is a matrix of true source signals or images. Each row of **Y** or **X** is a signal or 1D image representation, where *I* is a number of observed mixed signals and *J* is a number of hidden (source) components. The index *t* usually denotes a sample (discrete time instant), where *T* is the number of available samples. In BSS, we usually have *T* ≫ *I* ≥ *J*, and *J* is known or can be relatively easily estimated using SVD or PCA.

Our objective is to estimate the mixing matrix **A** and sources **X** subject to nonnegativity constraints of all the entries, given **Y** and possibly the knowledge on a statistical distribution of noisy disturbances.

Obviously, NMF is not unique in general case, and it is characterized by a scale and permutation indeterminacies. These problems have been addressed recently by many researchers [39–42], and in this paper, the problems will not be discussed. However, we have shown by extensive computer simulations that in practice with overwhelming probability we are able to achieve a unique nonnegative factorization (neglecting unavoidable scaling and permutation ambiguities) if data are sufficiently sparse and a suitable NMF algorithm is applied [43]. This is consistent with very recent theoretical results [40].

The noise distribution is strongly application-dependent, however, in many BSS applications, a Gaussian noise is expected. Here our considerations are restricted to this case, however, the alternative NMF algorithms optimized to different distributions of the noise exist and can be found, for example, in [37, 44, 45].

NMF was proposed by Paatero and Tapper [46, 47] but Lee and Seung [11, 48] highly popularized this method by using simple multiplicative algorithms to perform NMF. An extensive study on convergence of multiplicative algorithms for NMF can be found in [49]. In general, the multiplicative algorithms are known to be very slowly convergent for large-scale problems. Due to this reason, there is a need to search more efficient and fast algorithms for NMF. Many approaches have been proposed in the literature to relax these problems. One of them is to apply projected gradient (PG) algorithms [50–53] or projected alternating least-squares (ALS) algorithms [33, 54, 55] instead of multiplicative ones. Lin [52] suggested applying the Armijo rule to estimate the learning parameters in projected gradient updates for NMF. The NMF PG algorithms proposed by us in [53] also address the issue with selecting such a learning parameter that is the steepest descent and also keeps some distance to a boundary of the nonnegative orthant (subspace of real nonnegative numbers). Another very robust technique concerns exploiting the information from the second-order Taylor expansion term of a cost function to speed up the convergence. This approach was proposed in [45, 56], where the mixing matrix **A** is updated with the projected Newton method, and the sources in **X** are computed with the projected least-squares method (the fixed point algorithm).

In this paper, we extend our approach to NMF that we have initialized in [53]. We have investigated, extended, and tested several recently proposed PG algorithms such as the oblique projected Landweber [57], Barzilai-Borwein gradient projection [58], projected sequential subspace optimization [59, 60], interior-point Newton [61], and sequential coordinate-wise [62]. All the presented algorithms in this paper are quite efficient for solving large-scale minimization problems subject to nonnegativity and sparsity constraints.

The main objective of this paper is to develop, extend, and/or modify some of the most promising PG algorithms to a standard NMF problem and to find optimal conditions or parameters for such a class of NMF algorithms. The second objective is to compare the performance and complexity of these algorithms for NMF problems, and to discover or establish the most efficient and promising algorithms. We would like to emphasize that most of the discussed algorithms have not been implemented neither used till now or even tested before for NMF problems, but they have been rather considered for solving a standard system of algebraic equations: **Ax**(*k*) = **y**(*k*) (for only *k* = 1) where the matrix **A** and the vectors **y** are known. In this paper, we consider a much more difficult and complicated problem in which we have two sets of parameters and additional constraints of nonnegativity and/or sparsity. So it was not clear till now whether such algorithms would work efficiently for NMF problems, and if so, what kind of projected algorithms is the most efficient? To our best knowledge only the Lin-PG NMF algorithm is widely used and known for NMF problems. We have demonstrated experimentally that there are several novel PG gradient algorithms which are much more efficient and consistent than the Lin-PG algorithm.

In Section 2, we briefly discuss the PG approach to NMF. Section 3 describes the tested algorithms. The experimental results are illustrated in Section 4. Finally, some conclusions are given in Section 5.

## 2. Projected Gradient Algorithms

In contrast to the multiplicative algorithms, the class of PG algorithms has additive updates. The algorithms discussed here approximately solve nonnegative least squares (NNLS) problems with the basic alternating minimization technique that is used in NMF:

(1) $$\begin{array}{cc} \underset{x_{t}\geq 0}{\min}\,\;D_{F}(y_{t}||Ax_{t}) & =\;\frac{1}{2}∥y_{t}-Ax_{t}{∥}_{2}^{2},\;t\;=\;1,\ldots ,\;T, \end{array}$$

(2) $$\begin{array}{cc} \underset{{\underline{a}}_{i}\geq 0}{\min}\,\;D_{F}({\underline{y}}_{i}||X^{T}{\underline{a}}_{i}) & =\;\frac{1}{2}∥{\underline{y}}_{i}-X^{T}{\underline{a}}_{i}{∥}_{2}^{2},\;i\;=\;1,\ldots ,\;I \end{array}$$

or in the equivalent matrix form

(3) $$\begin{array}{cc} \underset{x_{jt}\geq 0}{\min}\,\;D_{F}(Y||AX) & =\;\frac{1}{2}∥Y-AX{∥}_{F}^{2}, \end{array}$$

(4) $$\begin{array}{cc} \underset{a_{ij}\geq 0}{\min}\,\;D_{F}(Y^{T}||X^{T}A^{T}) & =\frac{1}{2}∥Y^{T}-X^{T}A^{T}{∥}_{F}^{2}, \end{array}$$

where **A** = [**a**_1_, …, **a**_*J*_] ∈ ℝ^*I*×*J*^, $A^{T}=[{\underset{̲}{a}}_{1},\ldots ,{\underset{̲}{a}}_{I}]\in {\text{ℝ}}^{J\times I}$, **X** = [**x**_1_, …, **x**_*T*_] ∈ ℝ^*J*×*T*^, $X^{T}=[{\underline{x}}_{1},\ldots ,{\underline{x}}_{J}]\in {\text{ℝ}}^{T\times J}$, **Y** = [**y**_1_, …, **y**_*T*_] ∈ ℝ^*I*×*T*^, $Y^{T}=[{\underline{y}}_{1},\ldots ,{\underline{y}}_{I}]\in {\text{ℝ}}^{I\times T}$, and usually *I* ≥ *J*. The matrix **A** is assumed to be a full-rank matrix, so there exists a unique solution X* ∈ ℝ^*J*×*T*^ for any matrix **Y** since the NNLS problem is strictly convex (with respect to one set of variables {**X**}).

The solution **x**_t_* to (1) satisfies the Karush-Kuhn-Tucker (KKT) conditions:

(5) $$x_{t}^{*}\geq 0,\;\;g_{X}(x_{t}^{*})\geq 0,\;\;g_{X}{\left(x_{t}^{*}\right)}^{T}x_{t}^{*}\;=\;0,$$

or in the matrix notation

(6) $$X^{*}\geq 0,\;\;G_{X}(X^{*})\geq 0,\;\;\text{tr}\{G_{X}{\left(X^{*}\right)}^{T}X^{*}\}\;=\;0,$$

where **g**_*X*_ and **G**_*X*_ are the corresponding gradient vector and gradient matrix:

(7) $$\begin{array}{cc} g_{X}(x_{t}) & =\nabla_{x_{t}}D_{F}(y_{t}||Ax_{t})\;=\;A^{T}(Ax_{t}-y_{t}), \end{array}$$

(8) $$\begin{array}{cc} G_{X}(X) & =\nabla_{X}D_{F}(Y||AX)\;=\;A^{T}(AX-Y). \end{array}$$

Similarly, the KKT conditions for the solution ${\underline{a}}^{*}$ to (2), and the solution A* to (4) are as follows:

(9) $${\underset{̲}{a}}_{i}^{*}\geq 0,\;\;g_{A}({\underset{̲}{a}}_{i}^{*})\geq 0,\;\;g_{A}{\left({\underset{̲}{a}}_{i}^{*}\right)}^{T}{\underset{̲}{a}}_{i}^{*}\;=\;0,$$

or in the matrix notation:

(10) $$A^{*}\geq 0,\;\;G_{A}(A^{*})\geq 0,\;\;\text{tr}\{A^{*}G_{A}{\left(A^{*}\right)}^{T}\}\;=\;0,$$

where **g**_*A*_ and **G**_*A*_ are the gradient vector and gradient matrix of the objective function:

(11) $$\begin{array}{c} g_{A}({\underset{̲}{a}}_{t})=\nabla_{{\underset{̲}{a}}_{i}}D_{F}({\underset{̲}{y}}_{i}||X^{T}{\underset{̲}{a}}_{i})=(X^{T}{\underset{̲}{a}}_{i}-{\underset{̲}{y}}_{i}),\\ G_{A}(A)\;=\;\nabla_{A}D_{F}(Y^{T}||X^{T}A^{T})\;=\;(AX-Y)X^{T}. \end{array}$$

There are many approaches to solve the problems (1) and (2), or equivalently (3) and (4). In this paper, we discuss selected projected gradient methods that can be generally expressed by iterative updates:

(12) $$\begin{array}{ll} X^{\left(k+1\right)} & =\;P_{\Omega }[X^{\left(k\right)}-\eta_{X}^{\left(k\right)}P_{X}^{\left(k\right)}],\\ A^{\left(k+1\right)} & =\;P_{\Omega }[A^{\left(k\right)}-\eta_{A}^{\left(k\right)}P_{A}^{\left(k\right)}], \end{array}$$

where *P*_Ω_[*ξ*] is a projection of *ξ* onto a convex feasible set Ω = {*ξ* ∈ ℝ : *ξ * ≥ 0}, namely, the nonnegative orthant ℝ_+_ (the subspace of nonnegative real numbers), **P**_*X*_^(*k*)^ and **P**_*A*_^(*k*)^ are descent directions for **X** and **A**, and *η*_*X*_^(*k*)^ and *η*_*A*_^(*k*)^ are learning rules, respectively.

The projection *P*_Ω_[*ξ*] can be performed in several ways. One of the simplest techniques is to replace all negative entries in *ξ* by zero-values, or in practical cases, by a small positive number *ϵ* to avoid numerical instabilities. Thus,

(13) P[ξ] = max⁡{ϵ, ξ}.

However, this is not the only way to carry out the projection *P*_Ω_(*ξ*). It is typically more efficient to choose the learning rates *η*_*X*_^(*k*)^ and *η*_*A*_^(*k*)^ so as to preserve nonnegativity of the solutions. The nonnegativity can be also maintained by solving least-squares problems subject to the constraints (6) and (10). Here we present the exemplary PG methods that work for NMF problems quite efficiently, and we implemented them in the Matlab toolboxm, NMFLAB/NTFLAB, for signal and image processing [43]. For simplicity, we focus our considerations on updating the matrix **X**, assuming that the updates for **A** can be obtained in a similar way. Note that the updates for **A** can be readily obtained solving the transposed system **X** ^*T*^ **A** ^*T*^ = **Y** ^*T*^, having **X** fixed (updated in the previous step).

## 3. Algorithms

### 3.1. Oblique Projected Landweber Method

The Landweber method [63] performs gradient-descent minimization with the following iterative scheme:

(14) $$X^{\left(k+1\right)}=\;X^{\left(k\right)}-\eta G_{X}^{\left(k\right)},$$

where the gradient is given by (8), and the learning rate *η* ∈ (0, *η*_max_). The updating formula assures an asymptotical convergence to the minimal-norm least squares solution for the convergence radius defined by

(15) $$\eta_{\max}=\;\frac{2}{\lambda_{\max}(A^{T}A)},$$

where *λ*_max_(**A** ^*T*^ **A**) is the maximal eigenvalue of **A** ^*T*^ **A**. Since **A** is a nonnegative matrix, we have *λ*_max_(**A** ^*T*^ **A**) ≤ max_*j*_[**A** ^*T*^ **A** **1**_*J*_]_*j*_, where **1**_*J*_ = [1, …, 1]^*T*^ ∈ ℝ^*J*^. Thus the modified Landweber iterations can be expressed by the formula

(16) $$X^{\left(k+1\right)}=P_{\Omega }\left[X^{\left(k\right)}-\text{diag}\{\eta_{j}\}G_{X}^{\left(k\right)}\right],\;\text{where}\;\;\eta_{j}\;=\;\frac{2}{(A^{T}A1_{J}{)}_{j}}.$$

In the obliqueprojected Landweber (OPL) method [57], which can be regarded as a particular case of the PG iterative formula (12), the solution obtained with (14) in each iterative step is projected onto the feasible set. Finally, we have Algorithm 1 for updating **X**.

### 3.2. Projected Gradient

One of the fundamental PG algorithms for NMF was proposed by Lin in [52]. This algorithm, which we refer to as the Lin-PG, uses the Armijo rule along the projection arc to determine the steplengths *η*_*X*_^(*k*)^ and *η*_*A*_^(*k*)^ in the iterative updates (12). For the cost function being the squared Euclidean distance, **P**_*X*_ = (**A** ^(*k*)^)^*T*^ (**A** ^(*k*)^ **X** ^(*k*)^ − **Y**) and **P**_*A*_ = (**A** ^(*k*)^ **X** ^(*k*+1)^−**Y**)(**X** ^(*k*+1)^)^*T*^.

For computation of **X**, such a value of *η*_*X*_ is decided, on which

(17) $$\eta_{X}^{\left(k\right)}\;=\;\beta^{m_{k}},$$

where *m*_*k*_ is the first nonnegative integer *m* that satisfies

(18) $$D_{F}(Y\;||\;AX^{\left(k+1\right)})-D_{F}(Y\;||\;AX^{\left(k\right)})\leq \sigma \nabla_{X}D_{F}{\left(Y\;||\;AX^{\left(k\right)}\right)}^{T}(X^{\left(k+1\right)}-X^{\left(k\right)}).$$

The parameters *β* ∈ (0, 1) and *σ* ∈ (0,1) decide about a convergence speed. In this algorithm we set *σ* = 0.01, *β* = 0.1 experimentally as default.

The Matlab implementation of the Lin-PG algorithm is given in [52].

### 3.3. Barzilai-Borwein Gradient Projection

The Barzilai-Borwein gradient projection method [58, 64] is motivated by the quasi-Newton approach, that is, the inverse of the Hessian is replaced with an identity matrix **H**_*k*_ = *α*_*k*_ **I**, where the scalar *α*_*k*_ is selected so that the inverse Hessian has similar behavior as the true Hessian in the recent iteration. Thus,

(19) $$\begin{array}{c} X^{\left(k+1\right)}-X^{\left(k\right)}\approx \alpha_{k}(\nabla_{X}D(Y∥A^{\left(k\right)}X^{\left(k+1\right)})-\nabla_{X}D(Y∥A^{\left(k\right)}X^{\left(k\right)})). \end{array}$$

In comparison to, for example, Lin's method [52], this method does not ensure that the objective function decreases at every iteration, but its general convergence has been proven analytically [58]. The general scheme of the Barzilai-Borwein gradient projection algorithm for updating **X** is in Algorithm 2.

Since *D*_*F*_(**Y**||**AX**) is a quadratic function, the line search parameter **λ** ^(*k*)^ can be derived in the following closed-form formula:

(20) $$\lambda^{\left(k\right)}\;=\;\max\left\{0,\;\min\left\{1,\frac{\text{diag}\{{(\Delta }^{\left(k\right)}{)}^{T}\nabla_{X}D_{F}(Y||AX)\}}{\text{diag}\{{(\Delta }^{\left(k\right)}{)}^{T}A^{T}A\Delta^{\left(k\right)}\}}\right\}\right\}.$$

The Matlab implementation of the GPSR-BB algorithm, which solves the system **AX** = **Y** of multiple measurement vectors subject to nonnegativity constraints, is given in Algorithm 4 (see also NMFLAB).

### 3.4. Projected Sequential Subspace Optimization

The projected sequential subspace optimization (PSESOP) method [59, 60] carries out a projected minimization of a smooth objective function over a subspace spanned by several directions which include the current gradient and gradient from the previous iterations, and the Nemirovski directions. Nemirovski [65] suggested that convex smooth unconstrained optimization is optimal if the optimization is performed over a subspace which includes the current gradient **g**(**x**), the directions **d**_1_^(*k*)^ = **x** ^(*k*)^ − **x** ^(0)^, and the linear combination of the previous gradients **d**_2_^(*k*)^ = ∑_*n*=0_^*k*−1^ *w*_*n*_ **g**(**x**_*n*_) with the coefficients *w*_*n*_, *n* = 0, …, *k* − 1. The directions should be orthogonal to the current gradient. Narkiss and Zibulevsky [59] also suggested to include another direction: **p** ^(*k*)^ = **x** ^(*k*)^ − **x** ^(*k*−1)^, which is motivated by a natural extension of the conjugate gradient (CG) method to a nonquadratic case. However, our practical observations showed that this direction does not have a strong impact on the NMF components, thus we neglected it in our NMF-PSESOP algorithm. Finally, we have Algorithm 3 for updating **x**_*t*_ which is a single column vector of **X**.

The parameter *p* denotes the number of previous iterates that are taken into account to determine the current update.

The line search vector α_*_^(*k*)^ derived in a closed form for the objective function *D*_*F*_(**y**_*t*_||**A** **x**_*t*_) is as follows:

(21) $$\alpha_{*}^{\left(k\right)}=-((D^{\left(k\right)}{)}^{T}A^{T}AD^{\left(k\right)}+\lambda I{)}^{-1}(D^{\left(k\right)}{)}^{T}\nabla_{x_{t}}D_{F}(y_{t}||Ax_{t}).$$

The regularization parameter can be set as a very small constant to avoid instabilities in inverting a rank-deficient matrix in case that **D** ^(*k*)^ has zero-value or dependent columns.

### 3.5. Interior Point Newton Algorithm

The interior point Newton (IPN) algorithm [61] solves the NNLS problem (1) by searching the solution satisfying the KKT conditions (5) which equivalently can be expressed by the nonlinear equations

(22) $$D(x_{t})g(x_{t})\;=\;0,$$

where **D**(**x**_*t*_) = diag{*d*_1_(**x**_*t*_), …, *d*_*J*_(**x**_*t*_)}, **x**_*t*_ ≥ 0, and

(23) $$d_{j}(x_{t})=\{\begin{array}{ll} x_{jt} & \text{if}\;\;g_{j}(x_{t})\geq 0,\\ 1 & \text{otherwise}. \end{array}$$

Applying the Newton method to (22), we have in the *k*th iterative step

(24) $$(D_{k}(x_{t})A^{T}A\;+\;E_{k}(x_{t}))p_{k}\;=\;-D_{k}(x_{t})g_{k}(x_{t}),$$

where

(25) $$E_{k}(x_{t})\;=\;\text{diag}\{e_{1}(x_{t}),\ldots ,e_{J}(x_{t})\}.$$

In [61], the entries of the matrix **E**_*k*_(**x**_*t*_) are defined by

(26) $$e_{j}(x_{t})\;=\;\{\begin{array}{ll} g_{j}(x_{t}) & \text{if}\;\;0\leq g_{j}(x_{t})<\;x_{jt}^{\gamma },\;\;\text{or}\;\;(g_{j}(x_{t}){)}^{\gamma }>x_{jt},\\ 1 & \text{otherwise}, \end{array}$$

for 1 < *γ* ≤ 2.

If the solution is degenerate, that is, *t* = 1, …, *T*, ∃ *j* : *x*_jt_* = 0, and *g*_*jt*_=0, the matrix **D**_*k*_(**x**_*t*_)**A** ^*T*^ **A** + **E**_*k*_(**x**_*t*_) may be singular. To avoid such a case, the system of equations has been rescaled to the following form:

(27) $$W_{k}(x_{t})D_{k}(x_{t})M_{k}(x_{t})p_{k}\;=-W_{k}(x_{t})D_{k}(x_{t})g_{k}(x_{t})$$

with

(28) $$\begin{array}{ll} M_{k}(x_{t}) & =\;A^{T}A\;+\;D_{k}{\left(x_{t}\right)}^{-1}E_{k}(x_{t}),\\ W_{k}(x_{t}) & \;=\;\text{diag}\{w_{1}(x_{t}),\ldots ,\;w_{J}(x_{t})\},\\ w_{j}(x_{t}) & =\;(d_{j}(x_{t})\;+\;e_{j}(x_{t}){)}^{-1}, \end{array}$$

for **x**_*t*_ > 0. In [61], the system (27) is solved by the inexact Newton method, which leads to the following updates:

(29) $$\begin{array}{c} W_{k}(x_{t})D_{k}(x_{t})M_{k}(x_{t})p_{k}\;=\;-W_{k}(x_{t})D_{k}(x_{t})g_{k}(x_{t})\;+\;r_{k}(x_{t}), \end{array}$$

(30) p^k = max⁡{σ,1−∥PΩ[xt(k)+pk]−xt(k)∥2}(PΩ[xt(k)+pk]−xt(k)),

(31) xt(k+1) = xt(k)+ p^k,

where *σ* ∈ (0, 1), **r**_*k*_(**x**_*t*_) = **A** ^*T*^(**A** **x**_*t*_ − **y**_*t*_), and *P*_Ω_[·] is a projection onto a feasible set Ω.

The transformation of the normal matrix **A** ^*T*^ **A** by the matrix **W**_*k*_(**x**_*t*_)**D**_*k*_(**x**_*t*_) in (27) means the system matrix **W**_*k*_(**x**_*t*_)**D**_*k*_(**x**_*t*_)**M**_*k*_(**x**_*t*_) is no longer symmetric and positive-definite. There are many methods for handling such systems of linear equations, like QMR [66], BiCG [67, 68], BiCGSTAB [69], or GMRES-like methods [70], however, they are more complicated and computationally demanding than, for example, the basic CG algorithm [71]. To apply the CG algorithm the system matrix in (27) must be converted to a positive-definite symmetric matrix, which can be easily done with normal equations. The methods like CGLS [72] or LSQR [73] are therefore suitable for such tasks. The transformed system has the form

(32) $$\begin{array}{cc} Z_{k}(x_{t}){\overset{˜}{p}}_{k} & =-S_{k}(x_{t})g_{k}(x_{t})+\;{\overset{˜}{r}}_{k}(x_{t}), \end{array}$$

(33) $$\begin{array}{cc} S_{k}(x_{t}) & =\sqrt{W_{k}(x_{t})D_{k}(x_{t})}, \end{array}$$

(34) $$\begin{array}{ll} Z_{k}(x_{t}) & =S_{k}(x_{t})M_{k}(x_{t})S_{k}(x_{t})\\ & =S_{k}(x_{t})A^{T}AS_{k}(x_{t})+W_{k}(x_{t})E_{k}(x_{t}), \end{array}$$

with ${\overset{˜}{p}}_{k}\;=\;S_{k}^{-1}(x_{t})p_{k}$ and ${\tilde{r}}_{k}\;=\;S_{k}^{-1}(x_{t})r_{k}(x_{t})$.

Since our cost function is quadratic, its minimization in a single step is performed with combining the projected Newton step with the constrained scaled Cauchy step that is given in the form

(35) $$p_{k}^{\left(C\right)}=-\tau_{k}D_{k}(x_{t})g_{k}(x_{t}),\;\tau_{k}\;>\;0.$$

Assuming **x**_*t*_^(*k*)^ + **p**_*k*_^(*C*)^ > 0, *τ*_*k*_ is chosen as being either the unconstrained minimizer of the quadratic function *ψ*_*k*_(−*τ*_*k*_ **D**_*k*_(**x**_*t*_)**g**_*k*_(**x**_*t*_)) or a scalar proportional to the distance to the boundary along −**D**_*k*_(**x**_*t*_)**g**_*k*_(**x**_*t*_), where

(36) $$\begin{array}{ll} \psi_{k}(p) & =\frac{1}{2}p^{T}M_{k}(x_{t})p\;+\;p^{T}g_{k}(x_{t})\\ & =\frac{1}{2}p^{T}(A^{T}A\;+\;D_{k}^{-1}(x_{t})E_{k}(x_{t}))p+p^{T}A^{T}(Ax_{t}^{\left(k\right)}-y_{t}). \end{array}$$

Thus

(37) $$\tau_{k}\;=\;\{\begin{array}{c} \tau_{1}\;=\;\arg\;\underset{\tau }{\min}\,\;\psi_{k}(-\tau_{k}D_{k}(x_{t})g_{k}(x_{t}))\\ \;\;\;\;\;\;\;\,\;\;\text{if }\;\;x_{t}^{\left(k\right)}-\tau_{1}D_{k}(x_{t})g_{k}(x_{t})>\;0,\\ \tau_{2}\;=\;\theta \,\;\underset{j}{\min}\left\{\frac{x_{jt}^{\left(k\right)}}{(D_{k}(x_{t})g_{k}(x_{t}){)}_{j}}:(D_{k}(x_{t})g_{k}(x_{t}){)}_{j}>\;0\right\}\\ \;\;\;\;\;\;\;\;\;\;\;\;\;\;\text{otherwise}, \end{array}$$

where *ψ*_*k*_(−*τ*_*k*_ **D**_*k*_(**x**_*t*_)**g**_*k*_(**x**_*t*_)) = (**g**_*k*_(**x**_*t*_))^*T*^ **D**_*k*_(**x**_*t*_)**g**_*k*_(**x**_*t*_)/(**D**_*k*_ × (**x**_*t*_)**g**_*k*_(**x**_*t*_))^*T*^ **M**_*k*_(**x**_*t*_)**D**_*k*_(**x**_*t*_)**g**_*k*_(**x**_*t*_) with *θ* ∈ (0, 1). For *ψ*_*k*_(**p**_*k*_^(*C*)^) < 0, the global convergence is achieved if red(**x**_*t*_^(*k*+1)^ − **x**_*t*_^(*k*)^) ≥ *β*, *β* ∈ (0, 1) with

(38) $$\text{red}(p)\;=\;\frac{\psi_{k}(p)}{\psi_{k}(p_{k}^{\left(C\right)})}.$$

The usage of the constrained scaled Cauchy step leads to the following updates:

(39) st(k) = t(pk(C)−p^k)+p^k,xt(k+1) = xt(k)+st(k),

with *t* ∈ [0, 1), p^k and **p**_*k*_^(*C*)^ are given by (30) and (35), respectively, and *t* is the smaller square root (laying in (0,1)) of the quadratic equation:

(40) π(t) = ψk(t(pk(C)−p^k)+p^k)−βψk(pk(C)) = 0.

The Matlab code of the IPN algorithm, which solves the system **A** **x**_*t*_ = **y**_*t*_ subject to nonnegativity constraints, is given in Algorithm 5. To solve the transformed system (32), we use the LSQR method implemented in Matlab 7.0.

### 3.6. Sequential Coordinate-Wise Algorithm

The NNLS problem (1) can be expressed in terms of the following quadratic problem (QP) [62]:

(41) $$\underset{x_{t}\geq 0}{\min}\,\;\Psi (x_{t}),\;(t\;=\;1,\ldots ,T),$$

where

(42) $$\Psi (x_{t})=\;\frac{1}{2}x_{t}^{T}Hx_{t}\;+\;c_{t}^{T}x_{t},$$

with **H** = **A** ^*T*^ **A** and **c**_*t*_ = −**A** ^*T*^ **y**_*t*_.

The sequential coordinate-wise algorithm (SCWA) proposed first by Franc et al. [62] solves the QP problem given by (41) updating only single variable *x*_*jt*_ in one iterative step. It should be noted that the sequential updates can be easily done, if the function Ψ(**x**_*t*_) is equivalently rewritten as

(43) $$\begin{array}{ll} \Psi (x_{t}) & =\frac{1}{2}\underset{p\in ℐ}{\sum }\;\underset{r\in ℐ}{\sum }x_{pt}x_{rt}(A^{T}A{)}_{pr}\;+\underset{p\in ℐ}{\sum }x_{pt}(A^{T}y_{t}{)}_{pt}\\ & =\frac{1}{2}x_{jt}^{2}(A^{T}A{)}_{jj}\;+\;x_{jt}(A^{T}y_{t}{)}_{jt}\\ & \;+x_{jt}\underset{p\in ℐ\setminus \{j\}}{\sum }x_{pt}(A^{T}A{)}_{pj}\;+\;\underset{p\in ℐ\setminus \{j\}}{\sum }x_{pt}(A^{T}y_{t}{)}_{pt}\\ & \;+\frac{1}{2}\underset{p\in ℐ\setminus \{j\}}{\sum }\;\underset{r\in ℐ\setminus \{j\}}{\sum }x_{pt}x_{rt}(A^{T}A{)}_{pr}\\ & =\frac{1}{2}x_{jt}^{2}h_{jj}\;+\;x_{jt}\beta_{jt}\;+\;\gamma_{jt}, \end{array}$$

where *ℐ* = {1, …, *J*}, and

(44) $$\begin{array}{ll} h_{jj} & =\;(A^{T}A{)}_{jj},\\ \beta_{jt} & =\;(A^{T}y_{t}{)}_{jt}+\underset{p\in ℐ\setminus \{j\}}{\sum }x_{pt}(A^{T}A{)}_{pj}\\ & =\;[A^{T}Ax_{t}\;+\;A^{T}y_{t}{]}_{jt}-(A^{T}A{)}_{jj}x_{jt},\\ \gamma_{jt} & =\;\underset{p\in ℐ\setminus \{j\}}{\sum }x_{pt}(A^{T}y_{t}{)}_{pt}\;+\;\frac{1}{2}\underset{p\in ℐ\setminus \{j\}}{\sum }\;\underset{r\in ℐ\setminus \{j\}}{\sum }x_{pt}x_{rt}(A^{T}A{)}_{pr}. \end{array}$$

Considering the optimization of Ψ(**x**_*t*_) with respect to the selected variable *x*_*jt*_, the following analytical solution can be derived:

(45) $$\begin{array}{ll} x_{jt}^{*} & =\;\arg\;\min\Psi \left([x_{1t},\ldots ,x_{jt},\ldots ,x_{Jt}{]}^{T}\right)\\ & =\;\arg\;\min\frac{1}{2}x_{jt}^{2}h_{jj}\;+\;x_{jt}\beta_{jt}\;+\;\gamma_{jt}\\ & =\;\max\left(0,-\frac{\beta_{jt}}{h_{jj}}\right)\\ & =\;\max(0,\;x_{jt}-\frac{[A^{T}Ax_{t}{]}_{jt}\;+\;[A^{T}y_{t}{]}_{jt}}{(A^{T}A{)}_{jj}}). \end{array}$$

Updating only single variable *x*_*jt*_ in one iterative step, we have

(46) $$x_{pt}^{\left(k+1\right)}\;=\;x_{pt}^{\left(k\right)},\;\forall p\in ℐ\setminus \{j\},\;\;x_{jt}^{\left(k+1\right)}\,\;\neq \;x_{jt}^{\left(k\right)}.$$

Any optimal solution to the QP (41) satisfies the KKT conditions given by (5) and the stationarity condition of the following Lagrange function:

(47) $$ℒ(x_{t},\lambda_{t})=\frac{1}{2}x_{t}^{T}Hx_{t}\;+\;c_{t}^{T}x_{t}-\lambda_{t}^{T}x_{t},$$

where **λ**_*t*_ ∈ *R* ^*J*^ is a vector of Lagrange multipliers (or dual variables) corresponding to the vector **x**_*t*_. Thus, ∇_**x**_*t*__ *ℒ*(**x**_*t*_, **λ**_*t*_) = **H** **x**_*t*_ + **c**_*t*_ − **λ**_*t*_ = 0. In the SCWA, the Lagrange multipliers are updated in each iteration according to the formula

(48) $$\lambda_{t}^{\left(k+1\right)}\;=\;\lambda_{t}^{\left(k\right)}+\left(x_{jt}^{\left(k+1\right)}-x_{jt}^{\left(k\right)}\right)h_{j},$$

where **h**_*j*_ is the *j*th column of **H**, and **λ**_*t*_^(0)^ = **c**_*t*_.

Finally, the SCWA can take the following updates:

(49) $$\begin{array}{ll} x_{jt}^{\left(k+1\right)} & =\;\max\left(0,x_{jt}^{\left(k\right)}-\frac{\lambda_{j}^{\left(k\right)}}{(A^{T}A{)}_{jj}}\right),\\ x_{pt}^{\left(k+1\right)} & =\;x_{pt}^{\left(k\right)},\;\forall p\in ℐ\setminus \{j\}\\ \lambda_{t}^{\left(k+1\right)} & =\;\lambda_{t}^{\left(k\right)}+\left(x_{jt}^{\left(k+1\right)}-x_{jt}^{\left(k\right)}\right)h_{j}. \end{array}$$

## 4. Simulations

**Figure 1:**
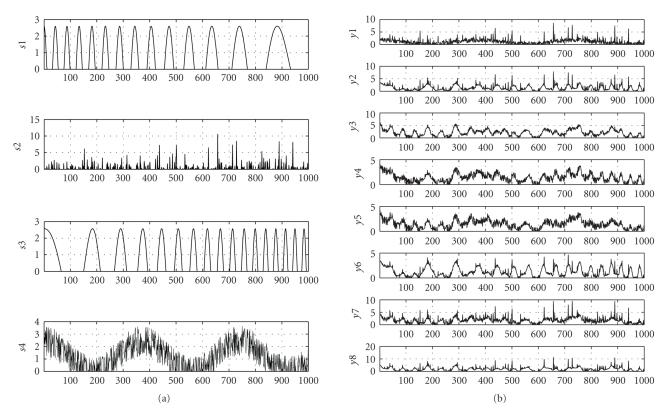
Dataset: (a) original 4 source signals, (b) observed 8 mixed signals.

All the proposed algorithms were implemented in our NMFLAB, and evaluated with the numerical tests related to typical BSS problems. We used the synthetic benchmark of 4 partially dependent nonnegative signals (with only *T* = 1000 samples) which are illustrated in Figure 1(a). The signals are mixed by random, uniformly distributed nonnegative matrix **A** ∈ ℝ^8×4^ with the condition number cond{**A**} = 4.11. The matrix **A** is displayed in

(50) $$A=\left[\begin{array}{cccc} 0.0631 & 0.7666 & 0.0174 & 0.6596\\ 0.2642 & 0.6661 & 0.8194 & 0.2141\\ 0.9995 & 0.1309 & 0.6211 & 0.6021\\ 0.2120 & 0.0954 & 0.5602 & 0.6049\\ 0.4984 & 0.0149 & 0.2440 & 0.6595\\ 0.2905 & 0.2882 & 0.8220 & 0.1834\\ 0.6728 & 0.8167 & 0.2632 & 0.6365\\ 0.9580 & 0.9855 & 0.7536 & 0.1703 \end{array}\right].$$

The mixing signals are shown in Figure 1(b).

Because the number of variables in **X** is much greater than in **A**, that is, *I* × *J* = 32 and *J* × *T* = 4000, we test the projected gradient algorithms only for updating **A**. The variables in **X** are updated with the standard projected fixed point alternating least squares (FP-ALS) algorithm that is extensively analyzed in [55].

In general, the FP-ALS algorithm solves the least-squares problem

(51) $$X^{*}\;=\;\arg\;\underset{X}{\min}\left\{\frac{1}{2}∥Y-AX{∥}_{F}^{2}\right\}$$

with the Moore-Penrose pseudoinverse of a system matrix, that is, in our case, the matrix **A**. Since in NMF usually *I* ≥ *J*, we formulate normal equations as **A** ^*T*^ **AX** = **A** ^*T*^ **Y**, and the least-squares solution of minimal *l*_2_-norm to the normal equations is **X**_LS_ = (**A** ^*T*^ **A**)^−1^ **A** ^*T*^ **Y** = **A** ^+^ **Y**, where **A** ^+^ is the Moore-Penrose pseudoinverse of **A**. The projected FP-ALS algorithm is obtained with a simple “half-rectified” projection, that is,

(52) $$X\;=\;P_{\Omega }\left[A^{+}Y\right].$$

The alternating minimization is nonconvex in spite of the cost function being convex with respect to one set of variables. Thus, most NMF algorithms may get stuck in local minima, and hence, the initialization plays a key role. In the performed tests, we applied the multistart initialization described in [53] with the following parameters: *N* = 10 (number of restarts), *K*_*i*_ = 30 (number of initial alternating steps), and *K*_*f*_ = 1000 (number of final alternating steps). Each initial sample of **A** and **X** has been randomly generated from a uniform distribution. Each algorithm has been tested for two cases of inner iterations, that is, with *k* = 1 and *k* = 5. The inner iterations mean a number of iterative steps that are performed to update only **A** (with fixed **X**, i.e., before going to the update of **X** and vice versa). Additionally, the multilayer technique [53, 54] with 3 layers (*L* = 3) is applied.

The multilayer technique can be regarded as multistep decomposition. In the first step, we perform the basic decomposition **Y** = **A**_1_ **X**_1_ using any available NMF algorithm, where **A**_1_ ∈ ℝ^*I*×*J*^ and **X**_1_ ∈ ℝ^*J*×*T*^ with *I* ≥ *J*. In the second stage, the results obtained from the first stage are used to perform the similar decomposition: **X**_1_ = **A**_2_ **X**_2_, where **A**_2_ ∈ ℝ^*J*×*J*^ and **X**_2_ ∈ ℝ^*J*×*T*^, using the same or different update rules, and so on. We continue our decomposition taking into account only the last achieved components. The process can be repeated arbitrary many times until some stopping criteria are satisfied. In each step, we usually obtain gradual improvements of the performance. Thus, our model has the form **Y** = **A**_1_ **A**_2_ ⋯ **A**_*L*_ **X**_*L*_ with the basis matrix defined as **A** = **A**_1_ **A**_2_ ⋯ **A**_*L*_ ∈ ℝ^*I*×*J*^. Physically, this means that we build up a system that has many layers or cascade connection of *L* mixing subsystems.

There are many stopping criteria for terminating the alternating steps. We stop the iterations if *s* ≥ *K*_*f*_ = 1000 or the following condition ||**A** ^(*s*)^ − **A** ^(*s*−1)^||_*F*_ < *ϵ* is held, where *s* stands for the number of alternating step, and *ϵ* = 10^−5^. Note that the condition (20) can be also used as a stopping criterion, especially as the gradient is computed in each iteration of the PG algorithms.

The algorithms have been evaluated with the signal-to-interference ratio (SIR) measures, calculated separately for each source signal and each column in the mixing matrix. Since NMF suffers from scale and permutation indeterminacies, the estimated components are adequately permuted and rescaled. First, the source and estimated signals are normalized to a uniform variance, and then the estimated signals are permuted to keep the same order as the source signals. In NMFLAB [43], each estimated signal is compared to each source signal, which results in the performance (SIR) matrix that is involved to make the permutation matrix. Let **x**_*j*_ and x^j be the *j*th source and its corresponding (reordered) estimated signal, respectively. Analogically, let **a**_*j*_ and a^j be the *j*th column of the true and its corresponding estimated mixing matrix, respectively. Thus, the SIRs for the sources are given by

(53) SIRj(X)=−20⁢ log⁡{∥x^j−xj∥2∥xj∥2}, j = 1,…, J,⁢  [dB]

and similarly for each column in **A** we have

(54) SIRj(A)=−20⁢ log⁡{∥a^j−aj∥2∥aj∥2}, j = 1,…, J,⁢  [dB].

We test the algorithms with the Monte Carlo (MC) analysis, running each algorithm 100 times. Each run has been initialized with the multistart procedure. The algorithms have been evaluated with the mean-SIR values that are calculated as follows:

(55) $$\begin{array}{cc} {\overset{¯}{\text{SIR}}}_{X} & =\frac{1}{J}\overset{J}{\underset{j=1}{\sum }}{\text{SIR}}_{j}^{\left(X\right)},\\ {\overset{¯}{\text{SIR}}}_{A} & =\frac{1}{J}\overset{J}{\underset{j=1}{\sum }}{\text{SIR}}_{j}^{\left(A\right)}, \end{array}$$

for each MC sample. The mean-SIRs for the worst (with the lowest mean-SIR values) and best (with the highest mean-SIR values) samples are given in Table 1. The number *k* means the number of inner iterations for updating **A**, and *L* denotes the number of layers in the multilayer technique [53, 54]. The notation *L* = 1 means that the multilayer technique was not used. The elapsed time [in seconds] is measured in Matlab, and it informs us in some sense about a degree of complexity of the algorithm.

For comparison, Table 1 contains also the results obtained for the standard multiplicative NMF algorithm (denoted as M-NMF) that minimizes the squared Euclidean distance. Additionally, the results of testing the PG algorithms which were proposed in [53] have been also included. The acronyms Lin-PG, IPG, RMRNSD refer to the following algorithms: projected gradient proposed by Lin [52], interior-point gradient, and regularized minimal residual norm steepest descent (the regularized version of the MRNSD algorithm that was proposed by Nagy and Strakos [74]). These NMF algorithms have been implemented and investigated in [53] in the context of their usefulness to BSS problems.

## 5. Conclusions

The performance of the proposed NMF algorithms can be inferred from the results given in Table 1. In particular, the results show how the algorithms are sensitive to initialization, or in other words, how easily they fall in local minima. Also the complexity of the algorithms can be estimated from the information on the elapsed time that is measured in Matlab.

It is easy to notice that our NMF-PSESOP algorithm gives the best estimation (the sample which has the highest best-SIR value), and it gives only slightly lower mean-SIR values than the Lin-PG algorithm. Considering the elapsed time, the PL, GPSR-BB, SCWA, and IPG belong to the fastest algorithms, while the Lin-PG and IPN algorithms are the slowest.

The multilayer technique generally improves the performance and consistency of all the tested algorithms if the number of observation is close to the number of nonnegative components. The highest improvement can be observed for the NMF-PSESOP algorithm, especially when the number of inner iterations is greater than one (typically, *k* = 5).

In summary, the best and the most promising NMG-PG algorithms are NMF-PSESOP, GPSR-BB, and IPG algorithms. However, the final selection of the algorithm depends on a size of the problem to be solved. Nevertheless, the projected gradient NMF algorithms seem to be much better (in the sense of speed and performance) in our tests than the multiplicative algorithms, provided that we can use the squared Euclidean cost function which is optimal for data with a Gaussian noise.

## Figures and Tables

**Algorithm 1:**
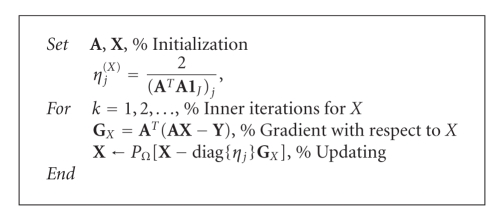
(OPL).

**Algorithm 2:**
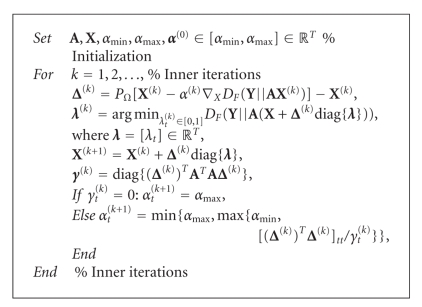
(GPSR-BB).

**Algorithm 3:**
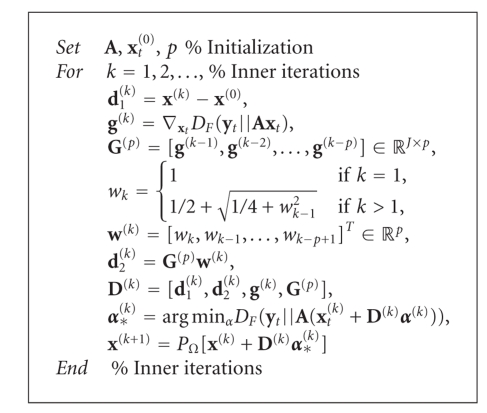
(NMF-PSESOP).

**Algorithm 4:**
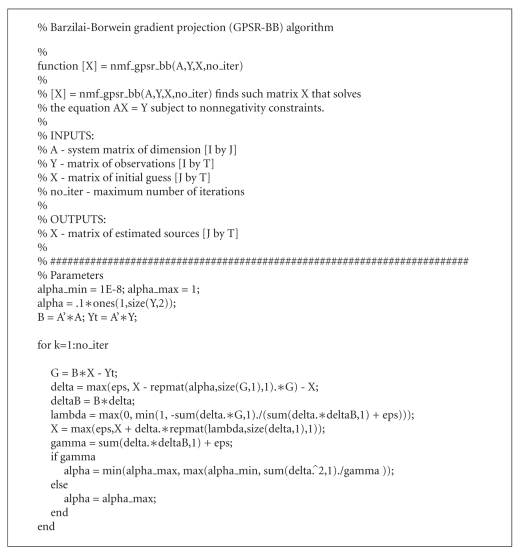


**Algorithm 5:**
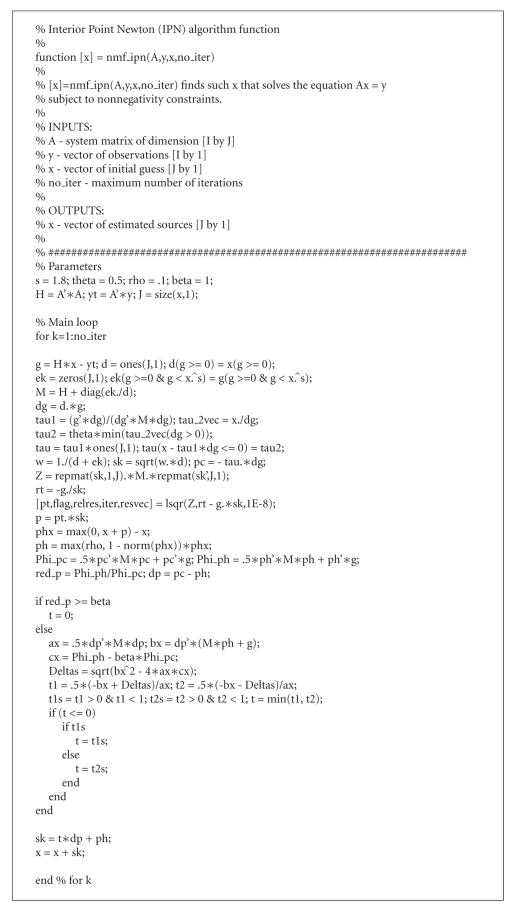


**Table 1:** Mean-SIRs [dB] obtained with 100 samples of Monte Carlo analysis for the estimation of sources and columns of mixing matrix from noise-free mixtures of signals in Figure 1. Sources **X** are estimated with the projected pseudoinverse. The number of inner iterations for updating **A** is denoted by *k*, and the number of layers (in the multilayer technique) by *L*. The notation *best* or *worst* in parenthesis that follows the algorithm name means that the mean-SIR value is calculated for the best or worst sample from Monte Carlo analysis, respectively. In the last column, the elapsed time [in seconds] is given for each algorithm with *k* = 1 and *L* = 1.

Algorithm	Mean-SIR_*A*_ [dB]				Mean-SIR_*X*_ [dB]				Time
	*L* = 1		*L* = 3		*L* = 1		*L* = 3		
	*k* = 1	*k* = 5	*k* = 1	*k* = 5	*k* = 1	*k* = 5	*k* = 1	*k* = 5	
M-NMF (best)	21	22.1	42.6	37.3	26.6	27.3	44.7	40.7	1.9
M-NMF (mean)	13.1	13.8	26.7	23.1	14.7	15.2	28.9	27.6	
M-NMF (worst)	5.5	5.7	5.3	6.3	5.8	6.5	5	5.5	
OPL(best)	22.9	25.3	46.5	42	23.9	23.5	55.8	51	1.9
OPL(mean)	14.7	14	25.5	27.2	15.3	14.8	23.9	25.4	
OPL(worst)	4.8	4.8	4.8	5.0	4.6	4.6	4.6	4.8	
Lin-PG(best)	36.3	23.6	78.6	103.7	34.2	33.3	78.5	92.8	8.8
Lin-PG(mean)	19.7	18.3	40.9	61.2	18.5	18.2	38.4	55.4	
Lin-PG(worst)	14.4	13.1	17.5	40.1	13.9	13.8	18.1	34.4	
GPSR-BB(best)	18.2	22.7	7.3	113.8	22.8	54.3	9.4	108.1	2.4
GPSR-BB(mean)	11.2	20.2	7	53.1	11	20.5	5.1	53.1	
GPSR-BB(worst)	7.4	17.3	6.8	24.9	4.6	14.7	2	23	
PSESOP(best)	21.2	22.6	71.1	132.2	23.4	55.5	56.5	137.2	5.4
PSESOP(mean)	15.2	20	29.4	57.3	15.9	34.5	27.4	65.3	
PSESOP(worst)	8.3	15.8	6.9	28.7	8.2	16.6	7.2	30.9	
IPG(best)	20.6	22.2	52.1	84.3	35.7	28.6	54.2	81.4	2.7
IPG(mean)	20.1	18.2	35.3	44.1	19.7	19.1	33.8	36.7	
IPG(worst)	10.5	13.4	9.4	21.2	10.2	13.5	8.9	15.5	
IPN(best)	20.8	22.6	59.9	65.8	53.5	52.4	68.6	67.2	14.2
IPN(mean)	19.4	17.3	38.2	22.5	22.8	19.1	36.6	21	
IPN(worst)	11.7	15.2	7.5	7.1	5.7	2	1.5	2	
RMRNSD(best)	24.7	21.6	22.2	57.9	30.2	43.5	25.5	62.4	3.8
RMRNSD(mean)	14.3	19.2	8.3	33.8	17	21.5	8.4	33.4	
RMRNSD(worst)	5.5	15.9	3.6	8.4	4.7	13.8	1	3.9	
SCWA(best)	12.1	20.4	10.6	24.5	6.3	25.6	11.9	34.4	2.5
SCWA(mean)	11.2	16.3	9.3	20.9	5.3	18.6	9.4	21.7	
SCWA(worst)	7.3	11.4	6.9	12.8	3.8	10	3.3	10.8	
